# Evidences from Clinical Trials in Down Syndrome: Diet, Exercise and Body Composition

**DOI:** 10.3390/ijerph17124294

**Published:** 2020-06-16

**Authors:** Rosa María Martínez-Espinosa, Mariola D Molina Vila, Manuel Reig García-Galbis

**Affiliations:** 1 Division of Biochemistry and Molecular Biology, Department of Agrochemistry and Biochemistry, Faculty of Sciences, University of Alicante, 03690 Alicante, Spain; rosa.martinez@ua.es; 2 Applied Biochemistry Research Group AppBiochem, University of Alicante, 03690 Alicante, Spain; mariola.Molina@ua.es; 3 Department of Mathematics, Faculty of Sciences, University of Alicante, 03690 Alicante, Spain; 4 Department of Nutrition and Dietetics, Faculty of Health Sciences, University of Atacama, Avda. Copayapu 2862, III Region, Copiapó 1530000, Chile; 5 Noncommunicable Diseases Research Group, Atacama 1410000, Chile

**Keywords:** Down’s syndrome, obesity, overweight, diet, exercise and body composition

## Abstract

Down syndrome (DS) is related to diseases like congenital heart disease, obstructive sleep apnea, obesity and overweight. Studies focused on DS associated with obesity and overweight are still scarce. The main objective of this work was to analyze the relationship between dietary intervention, physical exercise and body composition, in DS with overweight and obesity. This review is based on the PRISMA guidelines (Preferred Reporting Items for Systematic reviews and Meta-Analyses). Selection criteria for this analysis were: publications between January 1997 and December 2019; DS individuals with overweight and obesity; clinical trials using dietary intervention and physical exercise paying attention to changes in body composition. Selected clinical trials were focused on an exclusive intervention based on physical exercise. The anthropometric measures analyzed were body fat, BMI, waist circumference, body weight and fat free mass. The main conclusion is that prescribing structured physical exercise intervention may be related to a greater variation in body composition. Despite limited number of clinical trials analyzed, it can be assumed that the reported studies have not achieved optimal results and that the design of future clinical trials should be improved. Some guidelines are proposed to contribute to the improvement of knowledge in this field.

## 1. Introduction

Down Syndrome (DS) (OMIM#190685, Online Mendelian Inheritance in Man^®^, An Online Catalog of Human Genes and Genetic Disorders) is a genetic disorder caused by a trisomy of chromosome 21 and is the most common genetic cause of intellectual disability (ID) [1]. DS is associated with significant health problems as diseases such as congenital heart disease, obstructive sleep apnea, celiac disease and endocrinopathologies. Endocrine disorders are usually characterized by thyroid disorders, low bone mass, diabetes, short stature and propensity to be overweight/obese [1,2].

Life expectancy of people with DS has increased significantly from 12 years in 1949, to 60 years in 2004, and it is expected to increase in the near future [1,2]. Unfortunately, the increase in life expectancy is not parallel to the increase in the period of life with optimal health. Accelerated aging is identified in the case of DS based on two aspects: a. Clinical-pathological characteristics of the subject; b. Monitoring of molecular markers related to biological age and the aging process, highlighting the shortening of the telomeres [1]. Thus, several studies have connected the shortening of the telomeres with obesity, and particularly with the increase of BMI and adiposity causing accelerated aging [3,4].

Currently, there is a higher prevalence of overweight in ID patients (≥18 years) compared to those not affected by IDs, both in obesity (38.3% vs. 28%) and in morbid obesity (7.4% vs. 4.2%) [5]. Prevalence in overweight and obesity varied between 23–70% in DS patients (13.3–52.9 and 0–62.5%). Thus, young people with DS have higher rates of overweight and obesity than young people without DS [6]. 

The causes of the development of overweight and obesity in DS are: hypotonia (decreased muscle tone), susceptibility to systemic inflammation, metabolic diseases and/or slow metabolism [7]. Usually, people affected by DS consume less healthy food, and show physical limitations, depression, and lack of social and financial support. Besides, medications contribute to weight gain [8]. The key challenge for this field of knowledge in incoming years will be to identify therapeutic intervention strategies for weight loss that reduce body fat and systemic inflammation [6,9]. Therefore, there is a need to increase evidence-based clinical knowledge, with the aim of improving existing care programs [2,10,11]. 

Among the approaches used to monitor obesity and overweight, anthropometry reveals as one of the most efficient, cheaper and less time consuming. Anthropometry is considered a branch of biological anthropology; it is responsible for studying any physiological, psychological or anatomical trait. It is a powerful tool for the evaluation of nutritional status in the clinical environment and is used for general nutritional monitoring. Between the parameters measured, height and weight measurements are simple and quick. Other more complex measures include skin folds and circumferences, which require more professional training and involve different degrees of error [12]. To avoid errors or biases of these measurements, it is advisable to follow a protocol to measure these anthropometric variables, for example: the methodology ISAK (International Society for the Advancement of Cineanthropometry) [13,14,15]. 

In carrying out studies of an anthropometric nature, different methodologies have been developed during the last decades that have given rise to the following three groups: (i) Direct (dissection of corpses and neutron activation analysis); (ii) Indirect (hydrostatic densitometry; isotopic solution; total body potassium; DXA, dual X-ray absorptiometry; CT, computed tomography; QMR, quantitative magnetic resonance; ADP, air displacement plethysmography); (iii) Mixed or doubly indirect (Skin folds; BIA, bioelectrical impedance analysis and ultrasound) [16]. The measurement methods in the “Gold Standard” body composition to provide more information and to have greater accuracy are: full-body computerized tomography, four-compartment models (fat mass, muscle mass, other tissues and bone mass) and dual absorptiometry of X-rays. Here, accuracy is defined as the variation of body composition between different measurements in the same subject [17]. However, the most used methods in the clinical daily routine are: Skin folds; BIA and ultrasound [16,17].

Considering all the previous mentioned issues as well as the advantages of using anthropometric parameters to monitor nutritional status and obesity, the low number of studies about obesity and overweight in people with DS is striking. Consequently, this research is justified by the following facts:

1. DS individuals are characterized by high rates of overweight and obesity and low-quality life [1,6].
2. Obesity has negative health effects and lowers life expectancy [3,4].
3. Consumption of less healthy foods, lower levels of physical activity and medication are factors that enhance weight gain in DS [8].
4. There is a need to increase evidence-based clinical knowledge [2,10,11].

Thus, the main objective of this systematic review was to analyze the effectiveness of described interventions (based on dietary intervention and/or physical exercise) on the improvement of body composition and anthropometric parameters in people suffering from DS with overweight and obesity. As secondary objectives, the following have been identified: (i) To evaluate the methodological design quality of the CONSORT 2010 guidelines; (ii) To indicate the anthropometric parameters and units of measurement that record changes in body composition, in subjects with DS, overweight and obesity; (iii) Evaluation of body composition measuring instruments; (iv) To highlight dietary patterns and physical exercises that cause significant changes in the clinical trials analyzed. At the time of writing this work, the number of clinical studies performed involving the use of anthropometry to study DS individuals is low and criteria used are disparate. It is therefore necessary to carry out a detailed review of the methodology used, and the results achieved with a view to improving future interventions. From the results obtained in this analysis some guidelines are proposed to contribute to the improvement of knowledge in this field.

## 2. Methods

### 2.1. Search Strategy and Information Processing

This systematic review was conducted following the guidelines recommended by PRISMA (Preferred Reporting Items for Systematic reviews and Meta-Analyses) as well as the recommendations of the International Committee of Biomedical Journal Editors [18,19]. The information retrieval system “the Boolean model” was used [20]. 

**Figure 1:**
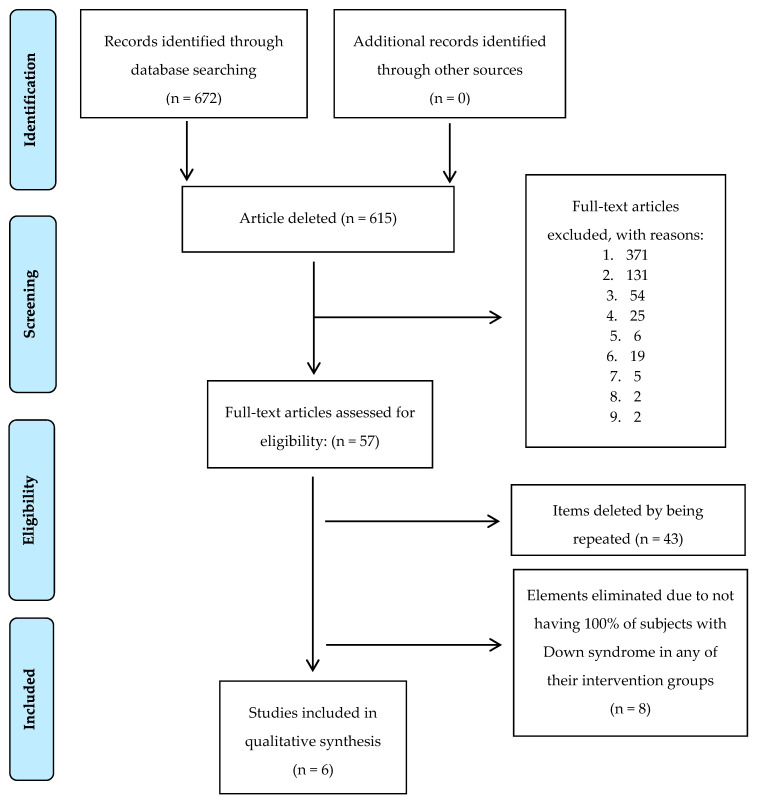
Flow chart in the screening process for the selection of included clinical trials [18]. Legend exclusion criteria: 1. Subjects not related to the variation of body composition in patients with excess weight; 2. Other intellectual or developmental disabilities that do not include Down syndrome; 3. Reviews and/or meta-analysis; 4. Interest in other pathologies (diabetes, hypertension, arthritis, heart failure, etc.); 5. Repeated articles; 6. Do not use dietary intervention or physical exercise as main treatments or use the help of pharmacological treatment, surgery or similar; 7. Animal intervention; 8. Languages other than English and Spanish; 9. Clinical trials involving children, adolescents and adults in the defined intervention groups.

The databases used were: PubMed (search engine for free access to the MEDLINE database of citations and abstracts of biomedical research articles); Scopus (bibliographic database containing abstracts and citations for academic journal articles); Web of Science (online scientific information service, provided by Thomson Reuters, integrated in ISI Web of Knowledge, WOK) (original articles based on clinical trials). The keywords were: intellectual disability, Down syndrome, overweight, obesity, diet and exercise. These keywords were obtained from the “MeSH database” (Medical Subject Heading) and NLM (The National Library of Medicine). Controlled vocabulary thesaurus was used for indexing articles for PubMed. The search strategies used are displayed in Table 1 and Figure 1.

In the “PubMed” database, the advanced form of search was used and the following options were selected “title/abstract”, specific date and languages; in “Scopus” the option of “document search”, “article title, abstract, keywords” and specific date; in “Web of Science”, the basic search was conducted and the following options were selected: theme, specific date and “article”.

Other details like date or language of the publications were not specified, and the search was conducted from January 1997 until December 2019. The open research questions at the beginning of this research were: What were the results obtained in the included clinical trials on the base of their design?; What are the anthropometric parameters and units of measurement registering changes in body composition, in subjects with DS, overweight and obesity?; What are the dietary and physical exercise patterns showing changes in body composition in the clinical trials analyzed?

### 2.2. Selection of the Articles Previously Identify

Each of the identified articles were independently analyzed by two researchers. PICOS strategy was used to define the eligibility criteria for this revision (population, intervention, comparisons, results and characteristics of clinical trials). Criteria included in the search strategy were: patients with DS or Trisomy 21 [21], who had been diagnosed in overweight or obese clinical trials; in the groups of children and adolescents, age will be less than or equal to 19 years [22], adults will be older than 19 years; clinical trial published between 1997 and 2019 in scientific journals in Spanish and English. These two languages were selected because they are two of three most spoken languages worldwide [23,24]. Besides, those patients involved in the selected studies had undergone an ID and/or physical exercise. Only the studies highlighting interest in the changes in body composition, through the anthropometric parameters and units of measurement have been included (Table 2 and Table 3) [25,26,27,28,29,30].

The exclusion criteria were patients showing other pathologies (diabetes mellitus, polycystic ovaries, etc.); clinical trials using pharmacology, gastric balloon and bariatric surgery; review articles and meta-analysis. In the selection of included clinical trials, those studies involving sample groups of all ages have been accepted to have a much broader view of the analysis. This has been a standard practice in previous reviews [31,32]. Data were extracted from the following 5 domains [33] (Table 2 and Table 3):

1. Population: characteristics of the population studied (country of origin, type of diagnostic criteria, number, age and gender), inclusion and exclusion criteria.
2. Interventions: exclusive and multidisciplinary as therapeutic treatments.
3. Comparators: inclusion of clinical trials, control and intervention groups are identified. In principle, only the intervention groups receive the therapeutic treatment that should cause changes in body composition.
4. Results: they are identified as variation in body composition, presenting significant and not significant variations.
5. Characteristics of clinical trials: authors, year of publication, type of clinical trial, duration of intervention, instrument of analysis of body composition, type of intervention used (exclusive or multidisciplinary) and body composition variation (measured with different anthropometric parameters and units of measurement).

### 2.3. Extraction of Data, Synthesis of Results and Risk of Loss of Information

Three tables and two figures were designed to perform this review. As first step, all the identified articles were listed including details about search engine and search strategy used. From these results, the included clinical trials in this work were selected (Figure 1 and Table 1). Clinical trials were grouped into several groups (Table 2 and Table 3), based on age and type of intervention. Three versions of each table were made. To minimize the risk of loss of information in the search of clinical trials, the term ID was added (Table 1). 

Table 2 and Table 3 were designed to record the characteristics of interest of the included clinical trials. The information collected was: author/s; study sample; duration of intervention; intervention/method; the variation of the body composition (body mass index, BMI; body fat, BF; fat-free mass, FFM; lean mass, LM; body weight, BW; waist circumference, WC) and the major changes in body composition. To identify the variation of these parameters and units, a Yes/No code has been used: YES, means that the article includes the analysis of the parameter expressed in its correspondent units and NO means that the article does not include the analysis of the parameter. Thus, the most significant changes body composition from each clinical trial was registered based on each parameter and unit (Table 2 and Table 3).

### 2.4. Use of the CONSORT 2010 Methodology

In order to analyze the quality in the design of clinical trials included in this work involving the highest variations in body composition, the *CONSORT 2010* method has been used (assessment and implementation guide on the most appropriate guidelines for the design of randomized trials) [34]. 

### 2.5. Evaluation of Body Composition Measuring Instruments

To evaluate how body composition has been measured, two groups have been established based on the degree of information about the instrument or their accuracy: (i) The level of information presented on the instruments used (model and its technical characteristics), as well as the monitoring of a protocol for the measurement of body composition (Table 4) (ii) The accuracy of the instruments has been assessed according to the scale of valuation indicated by Müller MJ and his colleagues [17] (p. 183).

For the first case, the following valuation scale is used (Table 4): (i) High (equivalent to three points, and reporting the type of instrument used and the measurement protocol followed); (ii) Moderate (equivalent to two points, and reporting the type of instrument used, but not the follow-up of any protocol); iii) Low (equivalent to a point, in this case neither the instrument nor the protocol used is indicated); (iv) Anthropometric parameter not measured (equivalent to zero points). The protocols used had to show some similarity with those presented in the aforementioned revisions [13,14,35,36].

## 3. Results 

### 3.1. Search Features and Types of Interventions Identified

Only 1% of the identified clinical trials have been included in this work to do the complete analysis. The Scopus database and the third search strategy provided most of the included trials (Figure 1 and Table 1).

Five clinical trials have been included in the study (6 papers, because Ordoñez and coworkers [a] and Ordoñez and coworkers [b] referred to the same study, but both have been included because they report some different parameters) (Table 2 and Table 3) [29,30]. Three of the studies involve children and adolescent populations (Table 2) and two of them work with adult patients (Table 3).

As a response to the second secondary objective, all of them were focused on an exclusive intervention based on physical exercise and the anthropometric measures analyzed were BF (in kg or %, 100%), BMI (in kg/m^2^, 80%), WC (in cm, 80%), BW (in kg, 60%) and FFM (in kg or %, 20%). A period of intervention between ten and twelve weeks was defined in all cases. 

### 3.2. Instruments for Measuring Body Composition

As it is displayed in Table 4, there are no significant differences in the level of information about the instruments used when evaluating children, adolescents and adults. Clinical trials that present more complete information and with less error/measurement bias (because they have followed a specific protocol) are those developed by Ordoñez and co-workers and Boer and co-workers [27,28]. 

For the evaluation of the instruments that measured fat mass, and in some cases fat-free mass, the precision scale indicated by Müller and colleagues was used, where the greatest precision was related to the smallest variation between the results when the measurement of body composition in a subject is repeated, and the lowest accuracy in the opposite case [17] (p. 183). Thus, the following groups were obtained: (i) The clinical trials of Seron, Boer and two of Ordoñez were the most accurate [25,28,29,30]; (ii) The other clinical trials used instruments of less precision [26,27]. The instruments used for measuring body fat were: (i) Bod Pod^®^ plethysmography equipment, body composition was calculated based on the equation of Siri (Life Measurement Inc., Concord, CA, USA) [25]; (ii) The percentage of fat mass was estimated through the summation of the four folds (triceps, biceps, subscapular and suprailiac regions) on the right side using the caliper de Lange and as the standard of reference to West’s rate and Deurenberg [26]; (iii) Percentage of fat mass was calculated from measurements according to the equation Durnin-Womersley using a Holtain lipometer (Holtain Ltd., Crymych, Dyfed, Wales, UK). [27]; (iv) Body composition was assessed by bioelectrical impedance analysis (Bodystat 1500 MDD, Douglas, Isle of Man, UK) [28]; (v) The percentage of fat mass was measured by bioelectrical impedance analysis (Tanita TBF521 bioelectrical) [29]; (vi) Fat-mass percentage was assessed by bioelectrical impedance analysis (Tanita TBF521) [30].

Considering the evaluations carried out, the third secondary objective is answered: most clinical trials about DS show a moderate level of information (it is not indicated whether or not a protocol has been followed in making measurements) and most instruments used to measure body fat and fat-free mass show a high level of accuracy.

### 3.3. Evaluation of the Design of Clinical Trials

In order to analyze the quality of the design of controlled clinical trials included in this work involving the highest variations in body composition (Seron and coworkers, Boer and coworkers and Ordoñez and coworkers) [25,28,29,30]. The CONSORT 2010 method has been used (assessment and implementation guide on the most appropriate guidelines for the design of randomized trials) [34]. Negative results should be considered for items that have not been performed or have been incompletely performed. Positive results are those in which the items were fulfilled entirely. Items related to the randomization process were eliminated for the analysis of the non-randomized trial [25]. 

As a response to the first secondary objective, positive results have been reported from half of the clinical trial analyzed. However, in no case, the percentage of positive responses was greater than 65%.

### 3.4. Changes in Body Composition in Down Syndrome: Children and Adolescents 

The three trials focused on children and adolescents were reported from Brazil, Chile and Spain, respectively [25,26,27]. Seron and coworkers, presented a quasi-experimental study in which the 41 patients (15.7 ± 2.7 years old) were assigned to two intervention groups plus a control group (*n* = 16, *n* = 15 and *n* = 10, respectively). The patients grouped in the intervention groups underwent two different types of training: aerobic (intensity of 50–70% of heart rate reserve 3 times per week) and resistance (12 maximum repetitions 2 times per week). The anthropometric measures analyzed were BW, BF, BMI and WC. Regarding to BW and BF, significant differences were not observed within the intervention groups nor between them. However, the differences were significant with respect to the control group and the two groups in BF but not in BW. The largest changes in BW occurred in the first group (0.7 kg), while in BF in the second one (0.5%). In BMI and WC, significant differences were observed within the first group (with changes of 0.5 kg/m^2^ and 1.1 cm, respectively), but not in the other one. Regarding BMI, there were significant differences in the second intervention group with respect to the other two groups, but these differences were already significant in the baseline values (Table 2). 

The other two articles presented interventions related to physical exercise in a single group of patients. Mosso and coworkers, reported an intervention in a group of 18 children between 5 and 9 years old. The intervention consisted in the application of a physical exercise program with different aerobic and resistance activities. The anthropometric measurements analyzed were BF, BMI and WC. BF increased and only significant differences in WC were obtained, with changes of around 3.07 cm. Ordoñez and coworkers applied a physical exercise intervention on a group of 22 male adolescents with a mean age of 16.2 ± 1.0 years. The training included exercises in water and land three times per week. The anthropometric measurements analyzed were BF and FFM (in kg and %) and BW. There were significant differences in BW and BF (changes of 3.6 kg in BW and 5.5 and 5.8% in BF) [27] (Table 2).

**Figure 2:**
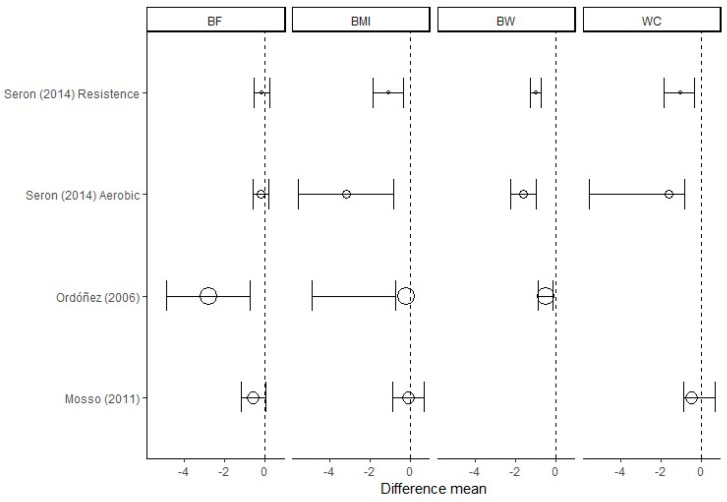
Within-group differences (with 95% confidence interval) in children and adolescents trials.

Regarding to BW (kg) and BF (kg and %), Ordoñez and coworkers reported the greatest changes in three months in a group from baseline, and the greatest variation was 3.6 kg, 5.5 kg and 5.8%, respectively [27]. It is important to highlight that the number of studies here included that follow inclusion and exclusion criteria was small. Consequently, these comparisons maybe are not significant and conclusive enough (Table 2 and Figure 2).

Considering the number of clinical trials analyzed, the fourth secondary objective is answered. The main finding regarding BMI (kg/m^2^) is that Seron and co-authors reported the greatest changes in three months and the greatest variation was 0.5 kg/m^2^ [25]; with respect to WC (cm), Mosso and coworkers, reported the greatest changes in three months and the greatest variation was 3.07 cm [26] (Table 2).

### 3.5. Changes in Body Composition in Down Syndrome: Adults 

Regarding to trials focused on adults, Boer and coworkers, described an experimental study located in the Republic of South Africa, in which the 42 patients (33.8 ± 8.6 years old, and 71.4% of female) were assigned to two interventions and a control group (*n* = 13, *n* = 13 and *n* = 16, respectively). The patients in the intervention groups underwent two different types of training: interval training (IT) versus continuous aerobic training (CAT). The anthropometric measures analyzed were BW, BF, BMI and WC. The significance of the differences within each group was not reported, but the differences between groups are not significant in BF and WC. On the contrary, in BW there are significant differences in the comparisons between the three groups, obtaining the greatest changes in the IT group (2.3 kg). In BMI no significant differences were observed between CAT groups and the control group, but between IT and the other two groups being the drop in this group of 0.8 kg/m^2^ (Table 3).

The other trial was reported by Ordoñez and coworkers, in which 21 females (between 18 and 30 years old) were randomly assigned to the intervention group (*n* = 11) participating in an aerobic training of three sessions per week, whilst the other 10 were assigned to a control group. The trial was conducted in Spain and the anthropometric measures analyzed were BF, BMI and WC. All measures showed significant changes in the intervention group, but the differences between the groups were not reported. Changes in the measures in the intervention group were 3.9%, 3.4 kg/m^2^ and 3.2 cm, respectively [29,30] (Table 3).

Considering the number of clinical trials analyzed, the fourth secondary objective is answered: regarding to BF (kg), Boer and coworkers, reported the greatest changes in three months and the greatest variation was 1.4 kg [28]; with respect to BF (%), BMI (kg/m^2^) and WC (cm), Ordoñez and coworkers, reported the greatest changes in two and a half months and the greatest variations were 3.9%, 3.4 kg/m^2^ and 3.2 cm, respectively [29]. Due to the small number of studies analyzed regarding this issue (2 works on DS in adults) the conclusions to be obtained may not be accurate and significant enough (Table 3). 

## 4. Discussion

Down syndrome is the most commonly occurring genetic chromosomal disorder and its estimated incidence is between 1 in 1000 to 1 in 1100 live births worldwide, according to the World Health Organization [37]. People suffering from DS are more likely to be overweight or obese than the general population [6]. Despite this, the number of studies (even clinical trials) on DS in connection with overweight or obesity is significantly low compared to other diseases. The lack of knowledge on this field justified from the very beginning this systematic review, which has been difficult to conduct because of the low number of clinical trials identified on this topic. Consequently, it has not been possible to address accurately the main objective. However, in view of the scarcity of knowledge in this area, it was decided to carry out this work, not only to identify what has been described so far, but also to highlight the need to deepen this field of research, from both levels basic and applied. Thus, three main limitations have been identified in this review: (i) Small number of studies based on clinical trials; (ii) Absence of clinical trials with dietary intervention; and (iii) Absence of studies based on multidisciplinary interventions. 

In order to analyze the information described so far on DS and overweight or obesity, the first approach was to evaluate the degree of information described in each study on the anthropometric measures carried out and the type of instrument used for the measurement. Regarding the level of information presented by each clinical trial on the measurements of body composition, it can be seen that in most clinical trials they are not following specific, standardized protocols for their measurement (Table 4) [13,14,35,36]. This information is of concern in clinical trials measuring body fat by measuring skin folds, because it is a method whose accuracy is lower due to errors resulting from measurement without a specific protocol [17] (p. 183). Among the instruments analyzed, the measuring tape and the skin fold register are sensitive measurement methods with variability in accuracy. It should therefore be stressed that in the future it is essential to use protocols for the different measurement techniques applied in order to ensure that the measures are reliable and reproducible [14,15,16]. In this sense, it is of special importance to identify two aspects: (i) The validity, precision and nature of the parameters that are monitored to estimate the body composition (without losing sight of both the daily and inter-individual variation); (ii) The variation of results according to the protocols of study used and the dynamics of weight changes (before, during and after the intervention) [38]. As a reflection, it is advisable to convey that despite the existence of more precise methods, it is possible to use any of the named methods if a protocol is used to decrease the variation in precision. In this way, changes in obesity can be monitored in response to the investment of time and money required for the monitoring.

In this review it has been found that the strategies that allowed greater changes in body composition, in children and adolescents were those base on planned physical exercise, considering the intensity, the duration, the number of repetitions, days per week and with programming by macrocycles, are the techniques that cause the greatest variation in body composition. The gradual increase of load in time and intensity is a technique that was also considered in two studies [25,26]. In the case of adults, the best results were observed when planned physical exercise was prescribed taking into consideration the intensity, duration, number of repetitions and days per week, including the continuous aerobic exercise [28,29]. In the absence of more evidences, the guidelines for dietary intervention and physical exercise in children, adolescents and adults with overweight or obesity without DS remain the main references to follow [39,40,41,42]. When prescribing physical exercise, it is recommended to use guidelines reporting positive results. These guidelines should follow the recommendations given in this review and by the American College of Sports Medicine (including degrees of evidence and greater accuracy in prescribing physical exercise) [43,44] (pp. 460, 1336).

In general terms, it can be concluded that there is a shortage of research personnel focused on this area of knowledge, as well as deficiencies in the means and infrastructures available for these studies. Therefore, research in this field, training of clinical staff in specific aspects of DD, and high-quality clinical care for subjects with this syndrome should be enhanced [10,45]. 

Regarding to the design of clinical assays involving DS individuals, it is worth mentioning that guidelines like CONSORT 2010 should be used [34] (p. 31), and studies involving individuals only suffering from DS has to be conducted [46] (pp. 137–140), so that dietary and multidisciplinary intervention is used to promote change in body composition. At the time of writing this review, most of the studies involved patients showing more diseases apart from DS, which makes difficult to establish correlations between changes of the measured parameters and exclusively DS.

As cited in the introduction and in Section 3.2, different type of instruments are used to measure key anthropometric parameters. Most instruments used to measure body fat and fat-free mass show a high level of accuracy in the analyzed studies, however, no clear protocols used to take the measurements are referred, which is a significant limitation of these reported studies. Apart from these instruments, the use of accelerometers as a precision method for evaluating physical activity is also suggested [47,48].

Among the issues to be considered in the design of further studies involving DS and obesity or overweight, the following should be included: cardiorespiratory fitness and analysis at molecular level. Cardiorespiratory fitness as a feasible method in subjects with DS without congenital heart disease. This approach is also useful to measure the level of physical condition in order to perform a safe physical activity [49]. Cardiorespiratory fitness is defined as the ability of the circulatory, respiratory and muscular systems to deliver oxygen during sustained physical activity. Records found in individuals with intellectual disabilities indicate a low level; levels in children, adolescents and men with intellectual disabilities are already low and decrease further with increasing age [50,51]. Regarding to the analysis at molecular level, it is important to mention that not only the shortening of the telomeres [1], but also other molecular targets like molecules sustaining systemic inflammation, hormones or peptides like leptin and adiponectin have to be considering to monitor obesity and overweight in DS. Recently it has been described that leptin is at higher concentration in young subjects with DS and adiponectin is higher in older subjects [52]. So, it is possible to establish correlations between leptin and adiponectin over time in DS as a tool to monitor weigh changes in people suffering from DS.

If all these objectives could be explored in the short term reaching positive results, it would be possible to assume that the monitoring of the variation of body composition through dietary intervention and physical exercise, could be an area of knowledge that contributes to the slowing down of aging in subjects with DS [1,2,3,4], allowing also the decrease of the high prevalence of overweight and obesity [6]. 

## 5. Conclusions

Down syndrome (DS) with overweight and obesity constitutes a field of knowledge in which research should continue with the aim of analyzing relationship between dietary intervention, physical exercise and body composition variation. From the results of this systematic review, it can be concluded that the studies carried out to date have not achieved optimal results and that the design of future clinical trials should be improved. Only reinforcing the knowledge related to this topic will improve daily clinical practice in sports sciences, nutrition and dietetics as well as in public health will improve. An increase in body composition variation will help to reduce the side effects of overweight and obesity, leading to a reduction in healthcare costs. 

## Figures and Tables

**Table 1:** Search strategies used to identify and to select clinical trials from January 1997 until December 2019.

	PubMed Identified/Included	Scopus Identified/Included	Web of Science Identified/Included	Summation Identified/Included
“Down’s syndrome” OR “Down syndrome” OR “cognitive disability” AND overweight OR obesity AND weight OR fat AND “physical activity” OR exercise OR weightlifting	21/4	47/7	85/6	153/17
“Down’s syndrome” OR “Down syndrome” OR “cognitive disability” AND overweight OR obesity AND weight OR fat AND feeding OR nutrition OR “nutritional counselling” or diet OR “dietary treatment”	25/2	64/4	44/3	133/9
“intellectual disability” or “Trisomy 21” AND overweight OR obesity AND weight OR fat AND “physical activity” OR exercise OR weightlifting	45/4	94/8	72/7	211/19
“intellectual disability” or “Trisomy 21” AND overweight OR obesity AND weight OR fat AND feeding OR nutrition OR “nutritional counselling” or diet OR “dietary treatment”	33/2	86/4	56/6	175/12
Summation Identified/Included	124/12	291/23	257/22	615/57

Articles or clinical trials identified: complete list of articles retrieved from various databases (PubMed, Scopus and Web of Science), without having made the selection of the clinical trials of interest. Articles or clinical trials included: articles that meet the selection criteria.

**Table 2:** Clinical trials including children and adolescents suffering DS in which body composition was monitored (intervention in physical exercise).

Reference [25,26,27]	Location	Study Design	Sample/Groups/Characteristics Studied	Intervention Method Characteristics Studied/Comparative Statistical Analysis of BC	Anthropometric Parameters (Statistical Results)					Variation in Body Composition Mean
					BW (kg/%)	BF (kg/%)	FFM (kg/%)	BMI (kg/m^2^/%)	WC (cm/%)	
[25]	South America (The Federative Republic of Brazil)	Clinical trial			YES (kg)	YES (%)	NO	YES (kg/m^2^)	YES (cm)	BW:
										IG1: −0.7 kg
			*n* = 41 (61% male)							IG2: −0.1 kg
				Comparative analysis:						CG: + 0.5 kg
			IG1, *n* = 16							
			IG2, *n* = 15	Within groups:						BF:
			CG, *n* = 10	IG1 (group aerobic training)	NS	NS		*p* < 0.05	*p* < 0.05	IG1: −0.2%
				IG2 (training group resistance)	NS	NS		NS	NS	IG2: −0.5%
			Age:	CG (control group)	NS	*p* < 0.05		NS	NS	CG: 2.7%
			IG1: 15.7 ± 2.7 years							
			IG2: 16 ± 2.8 years	Between groups						BMI:
			CG: 14.4 ± 2.5 years	IG1 vs. CG	NS	*p* < 0.05		NS	NS	IG1: −0.5 kg/m^2^
				IG2 vs. CG	NS	*p* < 0.05		*p* < 0.05	NS	IG2: −0.2 kg/m^2^
			Duration: 3 months	IG1 vs. IG2	NS	NS		*p* < 0.05	NS	CG: 0 kg/m^2^
										WC:
										IG1: −1.1 cm
										IG2: −0.1 cm
										CG: 0.5 cm
			[26]	South America (Chile)	Clinical trial	*n* = 18 (55.5% male)		Comparative analysis:	NO	YES (%)	NO	YES (kg/m^2^)	YES (cm)	BF: +0.18%
				
Age: 5–9 years							BMI: −0.16 kg/m^2^				
	NS	NS				*p* < 0.01					
Duration: 3 months							WC: −3.07 cm				
				
[27]	Europe (Spain)	Clinical trial			YES (kg)	YES (kg/%)	YES (kg/%)	NO	NO	BW: −3.6 kg
			*n* = 22 (100% male)							
				Comparative analysis:	*p* < 0.05	*p* < 0.05	NS			BF: −5.5 kg/−5.8%
			Age: 16.2 ± 1.0 years							
										FFM: +1.8 kg/+5.8%
			Duration: 3 months							

Body composition (BC); Down syndrome (DS); intervention group (IG); intellectual disability (ID); percentage (%); body mass index (BMI); body fat (BF); body weight (BW); fat-free mass (FFM); lean mass (LM); randomized clinical trial (RCT); waist circumference (WC); not significant (NS); the article includes the analysis of the parameter expressed in its correspondent units (YES); the article does not include the analysis of the parameter (NO); not specified (NE); minutes (min); week (wk); day (d); kilocalorie (Kcal).

**Table 3:** Clinical trials including adults suffering DS in which the variation of body composition was analyzed (intervention in physical exercise).

Reference [28,29,30]	Location	Study Design	Sample/Groups/Characteristics Studied	Intervention Method Characteristics Studied/Comparative Statistical Analysis of BC	Anthropometric Parameters (Statistical Results)					Variation in Body Composition Mean
					BW (kg/%)	BF (kg/%)	FFM (kg/%)	BMI (kg/m^2^/%)	WC (cm/%)	
[28]	Africa (Republic of South Africa)	RCT			YES (kg)	YES (kg/%)	NO	YES (kg/m^2^)	YES (cm)	BW:
										IG1: −2.3 kg
										IG2: −1 kg
										CG: +0.1 kg
				Comparative analysis:						
			*n* = 42 (71.4% female)							BF:
				Within groups:						IG1: −1.4 kg/−1.3%
			IG1: 13	IG1 (IT)						IG2: −1.2 kg/−0.8%
			IG2: 13	IG2 (CAT)	NE	NE		NE	NE	CG: +2.3 kg/−0.7%
			CG: 16	CG	NE	NE		NE	NE	
					NE	NE		NE	NE	BMI:
			Age: 33.8 ± 8.6 years	Between groups						IG1: −0.8 kg/m^2^
				IG1 vs. CG						IG2: −0.4 kg/m^2^
			Duration: 3 months	IG2 vs. CG	*p* < 0.05	NS		*p* < 0.05	NS	CG: −0.3 kg/m^2^
				IG1 vs. IG2	*p* < 0.05	NS		NS	NS	
					*p* < 0.05	NS		*p* < 0.05	NS	WC:
										IG1: −0.4 cm
										IG2: −1.3 cm
										CG: −1.4 cm
[29]	Europe (Spain)	RCT			NO	YES (%)	NO	YES (kg/m^2^)	YES (cm)	BF: −3.9%
			*n* = 20 (100% female)							
				Comparative statistical:						BMI: −3.4 kg/m^2^
			IG: 11							
			CG: 9	Within groups:						WC: −3.2 cm
				IG		*p* < 0.05		*p* < 0.05	*p* < 0.05	
			Age: 18–30 years	CG		NS		NS	NS	
			BMI: >30 kg/m^2^	Between groups		*p* < 0.05		*p* < 0.05	*p* < 0.05	
					
			Duration: 2.5 months							
[30]	Europe (Spain)	RCT			NO	YES (%)	NO	NO	YES (cm)	BF:
			*n* = 20 (100% female)							IG: −3.9%
				Comparative analysis:						CG: NE
			IG: 11							
			CG: 9	Within groups:						
				IG		*p* < 0.05			*p* < 0.05	WC:
			Age: 18–30 years	CG		NS			NS	IG: −3.2 cm
			BMI: >30 kg/m^2^	Between groups		*p* < 0.05			*p* < 0.05	CG: NE
				
			Duration: 2.5 months							

Down’s syndrome (DS); intervention group (IG); intellectual disability (ID); percentage (%); body mass index (BMI); body fat (BF); continuous aerobic training (CAT); fat-free mass (FFM); lean mass (LM); body weight (BW); randomized clinical trial (RCT); waist circumference (WC); not significant (NS); the article includes the analysis of the parameter expressed in its correspondent units (YES); the article does not include the analysis of the parameter (NO); not specified (NE); minutes (min); week (wk); day (d); kilocalorie (Kcal).

**Table 4:** Assessment of the level of information presented in the measurement of body composition.

Autor	BW	Height (BMI)	BF&FFM	WC	Individual Assessment (Mean)	General Evaluation (Mean)
Children and adolescents						
[25]	Moderate	Moderate	High	Moderate	Moderate (2.3)	
[26]	Moderate	Moderate	Moderate	High	Moderate (2.3)	Moderate (2.4)
[27]	Moderate	High	High	APNM	Moderate–High (2.7)	
Adults						
[28]	High	Moderate	High	High	Moderate–High (2.8)	
[29]	Moderate	Low	Moderate	Moderate	Low–Moderate (1.8)	Moderate (2.2)
[30]	APNM	Low	Moderate	High	Moderate (2)	

APNM: anthropometric parameter not measured; fat-free mass (FFM); body fat (BF); body weight (BW); randomized clinical trial (RCT); waist circumference (WC).

## References

[1] Franceschi C., Garagnani P., Gensous N., Bacalini M.G., Conte M., Salvioli S. (2019). Accelerated bio-cognitive aging in Down syndrome: State of the art and possible deceleration strategies. Aging Cell.

[2] Whooten R., Schmitt J., Schwartz A. (2018). Endocrine manifestations of Down syndrome. Curr. Opin. Endocrinol. Diabetes Obes..

[3] Kennedy B.K., Berger S.L., Brunet A., Campisi J., Cuervo A.M., Epel E.S., Franceschi C., Lithgow G.J., Morimoto R.I., Pessin J.E. (2014). Geroscience: Linking aging to chronic disease. Cell.

[4] Tzanetakou I.P., Katsilambros N.L., Benetos A., Mikhailidis D.P., Perrea D.N. (2012). Is obesity linked to aging?. Ageing Res. Rev..

[5] Hsieh K., Rimmer J.H., Heller T. (2013). Obesity and associated factors in adults with intellectual disability. J. Intellect. Disabil. Res..

[6] Bertapelli F., Pitetti K.H., Agiovlasitis S., Guerra-Junior G. (2016). Overweight and obesity in children and adolescents with Down syndrome—Prevalence, determinants, consequences, and interventions: A literature review. Res. Dev. Disabil..

[7] Brantmüller É., Gyuró M., Karácsony I. (2015). Development of Walking and Self-sufficiency Ability Related to Nutrition among People with Down Syndrome. Pract. Theory Syst. Educ..

[8] Cushing P., Spear D., Novak P., Rosenzweig L., Wallace L.S., Conway C., Wittenbrook W., Lemons S., Medlen J.G. (2012). Academy of Nutrition and Dietetics: Standards of Practice and Standards of Professional Performance for Registered Dietitians (Competent, Proficient, and Expert) in Intellectual and Developmental Disabilities. J. Acad. Nutr. Diet..

[9] Frasca D., Blomberg B.B. (2017). Adipose Tissue Inflammation Induces B Cell Inflammation and Decreases B Cell Function in Aging. Front. Immunol..

[10] Capone G., Chicoine B., Bulova P., Stephens M., Hart S.J., Crissman B., Videlefsky A., Myers K., Roizen N., Esbensen A. (2017). Co-occurring medical conditions in adults with Down syndrome: A systematic review toward the development of health care guidelines. Am. J. Med. Genet. Part A.

[11] Harris L., Melville C., Murray H., Hankey C. (2018). The effects of multi-component weight management interventions on weight loss in adults with intellectual disabilities and obesity: A systematic review and meta-analysis of randomised controlled trials. Res. Dev. Disabil..

[12] Ulijaszek S.J., A Kerr D. (1999). Anthropometric measurement error and the assessment of nutritional status. Br. J. Nutr..

[13] Madden A.M., Smith S. (2014). Body composition and morphological assessment of nutritional status in adults: A review of anthropometric variables. J. Hum. Nutr. Diet..

[14] Hume P., Marfell-Jones M. (2008). The importance of accurate site location for skinfold measurement. J. Sports Sci..

[15] Nádas J., Putz Z., Kolev G., Nagy S., Jermendy G. (2008). Intraobserver and interobserver variability of measuring waist circumference. Med. Sci. Monit..

[16] Gil A., Martínez de Victoria E., Maldonado J. (2010). Tratado de Nutrición.

[17] Müller M., Braun W., Pourhassan M., Geisler C., Bosy-Westphal A. (2015). Application of standards and models in body composition analysis. Proc. Nutr. Soc..

[18] Liberati A., Altman D.G., Tetzlaff J., Mulrow C., Gøtzsche P.C., Ioannidis J.P.A., Clarke M., Devereaux P.J., Kleijnen J., Moher D. (2009). The PRISMA Statement for Reporting Systematic Reviews and Meta-Analyses of Studies That Evaluate Health Care Interventions: Explanation and Elaboration. PLoS Med..

[19] The International Committee of Biomedical Journal Editors (ICMJE) (2017). Recommendations for the Preparation, Presentation, Editing and Publication of Academic Papers in Medical Journal. http://www.icmje.org/icmje-recommendations.pdf.

[20] Wiesman F., Hasman A., Herik H.V.D. (1997). Information retrieval: An overview of system characteristics. Int. J. Med. Inform..

[21] Ptomey L.T., Wittenbrook W. (2015). Position of the Academy of Nutrition and Dietetics: Nutrition Services for Individuals with Intellectual and Developmental Disabilities and Special Health Care Needs. J. Acad. Nutr. Diet..

[22] Rodd C.J., Metzger D., Sharma A.K., the Canadian Pediatric Endocrine Group (CPEG) Working Committee for National Growth Charts (2014). Extending World Health Organization weight-for-age reference curves to older children. BMC Pediatr..

[23] The 10 Most Spoken Languages in the World. Descarga en. https://danivoiceovers.com/en/los-10-idiomas-mas-hablados-mundo/.

[24] Los Idiomas, en Cifras: Cuántas Lenguas Hay en el Mundo? Europapress. Descarga en. https://www.europapress.es/sociedad/noticia-idiomas-cifras-cuantas-lenguas-hay-mundo-20190221115202.html.

[25] Seron B.B., Silva R.A., Greguol M. (2014). Effects of two programs of exercise on body composition of adoles-cents with Down syndrome. Rev. Paul. Pediatr..

[26] Constanza Mosso C., Patricia Santander V., Paulina Pettinelli R., Marcela Valdés G., Magdalena Celis B., Fabián Espejo S. (2011). Evaluation of a physical activity intervention among children with down’s syndrome. Rev. Chil. Pediatr..

[27] Ordonez F.J., Rosety M., Rosety-Rodriguez M. (2006). Influence of 12-week exercise training on fat mass per-centage in adolescents with Down syndrome. Med. Sci. Monit..

[28] Boer P.-H., Moss S.J. (2016). Effect of continuous aerobic vs. interval training on selected anthropometrical, physiological and functional parameters of adults with Down syndrome. J. Intellect. Disabil. Res..

[29] Ordonez F.J., Fornieles G., Rosety M.A., Rosety I., Diaz A.J., Camacho A. (2013). A Short Training Pro-gram Reduced Fat Mass and Abdominal Distribution in Obese Women with Intellectual Disability. Int. J. Morphol..

[30] Ordonez F.J., Fornieles-Gonzalez G., Camacho A., Rosety M.A., Rosety I., Diaz A.J., Rodríguez J.R. (2012). Anti-inflammatory effect of exercise, via reduced leptin levels, in obese women with Down syndrome. Int. J. Sport Nutr. Exerc. Metab..

[31] Ogg-Groenendaal M., Hermans H., Claessens B. (2014). A systematic review on the effect of exercise interventions on challenging behavior for people with intellectual disabilities. Res. Dev. Disabil..

[32] Shin I.-S., Park E.-Y. (2012). Meta-analysis of the effect of exercise programs for individuals with intellectual disabilities. Res. Dev. Disabil..

[33] Es L.R.R., Resende E.S., Diniz A.L.D., Penha-Silva N., O’Connell J.L., Gomes P.F.S., Zanetti H.R., Roerver-Borges A.S., Veloso F.C., De Souza F.R. (2018). Epicardial adipose tissue and metabolic syndrome. Medicine.

[34] Moher D., Hopewell S., Schulz K.F., Montori V.M., Gøtzsche P.C., Devereaux P.J., Elbourne D., Egger M., Altman U.G. (2012). CONSORT 2010 explanation and elaboration: Updated guidelines for reporting parallel group randomised trials. Int. J. Surg..

[35] Fields D.A., Higgins P.B., Radley D. (2005). Air-displacement plethysmography: Here to stay. Curr. Opin. Clin. Nutr. Metab. Care.

[36] Kyle U.G., Bosaeus I., De Lorenzo A.D., Deurenberg P., Elia M., Gómez J.M., Heitmann B.L., Kent-Smith L., Melchior J.-C., Pirlich M. (2004). Bioelectrical impedance analysis-part II: Utilization in clinical practice. Clin. Nutr..

[37] WHO Human Genomics in Global Health. https://www.who.int/genomics/public/geneticdiseases/en/index1.html.

[38] Müller M., Bosy-Westphal A. (2019). Effect of Over- and Underfeeding on Body Composition and Related Metabolic Functions in Humans. Curr. Diabetes Rep..

[39] Bray G., E Heisel W., Afshin A., Jensen M.D., Dietz W.H., Long M.W., Kushner R.F., Daniels S.R., A Wadden T., Tsai A.G. (2018). The Science of Obesity Management: An Endocrine Society Scientific Statement. Endocr. Rev..

[40] Garvey W.T., Mechanick J.I., Brett E.M., Garber A.J., Hurley D.L., Jastreboff A.M., Nadolsky K., Pessah-Pollack R., Plodkowski R. (2016). American Association of Clinical Endocrinologist and American College of Endocrinology Comprehensive Clinical Practice Guidelines for Medical Care of Patients with obesity. Endocr. Pract..

[41] Yumuk V., Tsigos C., Fried M., Schindler K., Busetto L., Micic A., Toplak H., Obesity Management Task Force of the European Association for the Study of Obesity (2015). European Guidelines for Obesity Management in Adults. Obes. Facts.

[42] Fitch A., Fox C., Bauerly K., Gross A., Heim C., Judge-Dietz J., Kaufman T., Krych E., Kumar S., Landin D. Prevention and Management of Obesity for Children and Adolescents. Institute for Clinical Systems Improvement. https://www.healthpartners.com/ucm/groups/public/@hp/@public/documents/documents/cntrb_037112.pdf.

[43] Garber C.E., Blissmer B., Deschenes M.R., Franklin B.A., Lamonte M.J., Lee I.M. (2011). American Col-lege of Sports Medicine position stand. Quantity and quality of exercise for developing and maintaining cardiorespiratory, musculoskeletal, and neuromotor fitness in apparently healthy adults: Guidance for prescribing exercise. Med. Sci. Sports Exerc..

[44] Donnelly J.E., Blair S.N., Jakicic J.M., Manore M.M., Rankin J.W., Smith B.K. (2009). Appropriate Physical Activity Intervention Strategies for Weight Loss and Prevention of Weight Regain for Adults. Med. Sci. Sports Exerc..

[45] Grondhuis S.N., Aman M.G. (2013). Overweight and obesity in youth with developmental disabilities: A call to action. J. Intellect. Disabil. Res..

[46] Argimon J.M., Jimenéz J. (2013). Métodos de Investigación Clínica y Epidemiológica.

[47] Pitchford E.A., Adkins C., Hasson R.E., Hornyak J.E., Ulrich D. (2018). Association between Physical Activity and Adiposity in Adolescents with Down Syndrome. Med. Sci. Sports Exerc..

[48] Loyen A., Clarke-Cornwell A., Anderssen S.A., Hagströmer M., Sardinha L.B., Sundquist K., Ekelund U., Steene-Johannessen J., Baptista F., Hansen B.H. (2016). Sedentary Time and Physical Activity Surveillance Through Accelerometer Pooling in Four European Countries. Sports Med..

[49] Pastore E., Marino B., Calzolari A., Digilio M.C., Giannotti A., Turchetta A. (2000). Clinical and cardiorespiratory assessment in children with Down syndrome without congenital heart disease. Arch. Pediatr. Adolesc. Med..

[50] Carvalho T., Massetti T., Da Silva T.D., Crocetta T.B., Guarnieri R., De Abreu L.C., Monteiro C.B.D.M., Garner D.M., Ferreira C. (2018). Heart rate variability in individuals with Down syndrome—A systematic review and meta-analysis. Auton. Neurosci..

[51] Oppewal A., Hilgenkamp T.I.M., Van Wijck R., Evenhuis H.M. (2013). Cardiorespiratory fitness in individuals with intellectual disabilities—A review. Res. Dev. Disabil..

[52] Nixon D. (2018). Down Syndrome, Obesity, Alzheimer’s Disease, and Cancer: A Brief Review and Hypothesis. Brain Sci..

